# Maternal Characteristics and U.S. Prenatal Care: Associations with Neonatal Health and Postpartum Maternal Wellbeing

**DOI:** 10.1007/s10995-025-04128-0

**Published:** 2025-07-07

**Authors:** Inga Nordgren, Robert J. Duncan, Kameron J. Moding, German E. Posada

**Affiliations:** 1 Department of Human Development and Family Science, Purdue University, West Lafayette, Indiana, United States; 2 Department of Human Development and Family Science, Colorado State University, Boulder, Colorado, United States

**Keywords:** Maternal characteristics, Prenatal care, Neonatal health, Postpartum wellbeing

## Abstract

Prenatal care provides an opportunity for the promotion of healthy parent and neonatal outcomes, but there are gaps in understanding the consequences and antecedents of prenatal care experiences during pregnancy. The objective of the current study was to provide insight into the mediational processes of sociodemographic and pregnancy characteristics on neonatal health and postpartum maternal wellbeing outcomes through prenatal care experiences. Data were analyzed from the publicly available *Listening to Mothers III* dataset comprised of 2,400 online-survey participants (ages 18–45) who gave birth to singleton infants in hospitals between July 2011 and June 2012 in the U.S. Primary analyses were conducted using two separate path analysis models examining maternal characteristics (i.e., sociodemographic and pregnancy factors) and the mediation of prenatal care experiences (i.e., responsive provider behavior, week of first prenatal visit, and attendance in group prenatal care) on infant health (i.e., preterm, low birthweight, NICU stays) and postpartum maternal wellbeing (i.e., social support, depression, confidence). The perceived need for treatment of depression and smoking during pregnancy resulted in 0.15 and 0.20 larger proportions of infants admitted into the NICU, respectively. Women who reported responsive provider behavior indicated higher social support (*b* = 0.29, *p* <.001), less depressive symptomology (*b* = -0.20, *p* <.001), and felt more confident (*b* = 0.07, *p* =.005). While maternal characteristics were related to neonatal health, the interpersonal experiences during prenatal care related to postpartum maternal wellbeing, indicating a need for providers to bolster their responsiveness to women during prenatal visits.

**Supplementary Information:**

The online version contains supplementary material available at 10.1007/s10995-025-04128-0.

## Significance

Prenatal care is a widely utilized health intervention during pregnancy which primarily focuses on neonatal health. However, the prenatal care experiences in this study (i.e., reported provider behavior, gestational week of first visit, participation in group care) were not associated with improved infant birth outcomes. Instead, women who reported responsive provider behavior and who engaged with group prenatal care had more positive rates of postpartum wellbeing. These findings suggest that interpersonal experiences during prenatal care have associations lasting beyond birth and delivery, and thus more focus should be given to enhancing the patient-provider relationship and support resources during pregnancy.

**Supplementary Information:**

The online version contains supplementary material available at 10.1007/s10995-025-04128-0.

Maternal and infant health continues to be a compelling and urgent topic in the United States. Fortunately, prenatal care is often the first opportunity for prevention, intervention, and the promotion of healthy mother and child outcomes given that 95% of pregnant women in the U.S. receive prenatal care (Currie & Rossin-Slater, 2015). However, the quality of care and the barriers to care remain a point of concern. There are gaps in understanding the consequences and antecedents of prenatal care experiences during pregnancy. By targeting several specific factors related to prenatal care (i.e., reported provider behavior, gestational week of first visit, participation in group care), this study hopes to improve the understanding of how these experiences are both predicted by various sociodemographic backgrounds and pregnancy characteristics of individuals and are associated with neonatal health and postpartum parent wellbeing through mediation models.

The intent of the current study is twofold. First, it attempts to identify who would most significantly benefit from improved care practices and what that care might look like (e.g., responsive provider training, structural changes to accommodate duration of prenatal care visits, promoting accessibility to early entry into prenatal care, and encouraging the use of group prenatal care). Second, it examines to what extent the differences in neonatal health and postpartum maternal wellbeing outcomes persist beyond prenatal care factors. This information has the potential to benefit family scholars, medical providers, and prenatal care policymakers in prioritizing equitable care practices.

The topic is important for two major reasons. One, adverse neonatal outcomes have potential life-long consequences. For infants, this can manifest as poor physical health, negative medical outcomes, or issues with developmental delays (Ickovics et al., 2007; Larroque et al., 2008; Saigal & Doyle, 2008). For new parents, disadvantageous adjustment to parenthood may look like unstable social support (Prabhakar et al., 2019), postpartum depression (Conroy et al., 2012), or lack of parental confidence (Amin et al., 2018). The other reason this topic is important is because of the increased need for birth equity among the pregnant population. With the diversity of sociodemographic backgrounds, there are significant differences in the ways in which pregnant individuals are treated within the health care system (Attanasio & Kozhimannil, 2015; Bryne & Tanesini, 2015; Finocharo et al., 2021; Goddu et al., 2015; Ward et al., 2013; Xaverius et al., 2018). Targeting specific factors related to prenatal care can improve the understanding of how these experiences relate to neonatal health and new parent wellbeing.

## Theoretical Frameworks

The current study follows the Health Equity Framework (HEF) developed by Peterson and colleagues (2021) that promotes equity of health outcomes by considering the socioecological and life-course perspective of the target population. Health equity directly ties to health disparities. Defined by Braveman (2014), health disparities are “a specific set of health differences that raise concerns about social justice, because they adversely affect groups of people who are economically and/or socially disadvantaged” (p. 367). These disparities can be a result of both biological and social determinants of health (e.g., education, income, marital status, etc.) and can disproportionally affect groups based on their race or ethnicity (Braveman, 2014; Price et al., 2010). Recognizing that many individual-level interventions do not adequately address the contexts behind prevalent health disparities, the HEF considers multilevel-based interactions akin to Bronfenbrenner’s ecological system (1997) where an individual’s interactions with agents, groups, and institutions in their environment are considered.

The life-course perspective acknowledges differences in individual, relational, historical, and systemic experiences across the life span (Elder, 1998; Peterson et al., 2021) and is crucial in developing interventions to alleviate health disparities that remain across decades (Braveman, 2014). Described by Baumert and colleagues (2012) as the process by which “relatively small initial advantages lead to a disproportionate accumulation of personal, social, and economic resources across the lifespan” (p. 1348), the concept of cumulative advantage may help explain the growing inequities between individuals and social groups. Conversely, cumulative disadvantage, then, is not based on merit but on the systematic (un)availability of resources and opportunities (Dannefer, 2003). The life-course is a particularly important perspective given the stark and persistent infant and maternal health disparities among racial groups in the United States (Lu & Halfon, 2003). Although Lu and Halfon (2003) note some hesitancies about the impact of a nine-month prenatal intervention reversing a lifespan worth of developmental experiences, they acknowledge that the life-course perspective regarding health of an infant may, in fact, start with prenatal care.

### Antecedents and Consequences of Prenatal Care Experiences

With over 12 million prenatal care visits occurring in a year in the U.S. (Santo & Kang, 2023), it is widely considered that prenatal care has the potential to impact neonatal and infant health. In fact, those who do not receive adequate prenatal care, or no prenatal care at all, can be up to eight times more likely to have adverse pregnancy outcomes (Shah et al., 2018; Xaverius et al., 2016). However, adequate prenatal care is defined based on the week of entry into prenatal care and the number of sessions an individual attends over their pregnancy (Kotelchuck, 1994). This definition measures quantity over quality. It does not consider the sociodemographic background of pregnant individuals who may face systemic barriers to initiating and continuing care, the quality of the patient-provider relationship, or the content or utility of the prenatal care sessions. These considerations (or lack of) are known issues. Insurance status and age are cited as barriers to care and perceived causes of discrimination during pregnancy (Attanasio & Kozhimannil, 2015; Ward et al., 2013). Further, according to an analysis of the National Survey of Family Growth data from 2017 to 2019, only about 25% of all women rated their family planning provider as “excellent” when it came to communication and information, with Black and Hispanic women being the least likely to endorse their provider as such (Finocharo et al., 2021). Research also indicates that pregnant individuals desire more parental knowledge and social support embedded in prenatal care (Chae et al., 2017; Kanotra et al., 2007; McDonald et al., 2016).

Some action has been taken to address these experiences during prenatal care, like the development of group prenatal care (GPC) as a promising, equitable alternative to individual care (Chae et al., 2017; Crockett et al., 2019; Ickovics et al., 2007). Group sessions last an average of two hours and consist of individual health assessments, pregnancy-related educational components, and social support through the relationships formed between parents in the group and its providers. Yet it is still difficult to know how these system-level experiences operate between pertinent individual-level characteristics and outcomes at birth and postpartum. As such, this study aims to provide insight into the mediational processes of sociodemographic and pregnancy characteristics on neonatal health and postpartum maternal wellbeing outcomes through several prenatal care experiences.

In this study, we include sociodemographic characteristics that are relevant for maternal and neonatal health disparities (i.e., age, race, parity, income, insurance status, marital status, educational level; Fahey & Shenassa, 2013; Peterson et al., 2021). Additionally, we include smoking and depression during pregnancy as antecedent factors to both experiences in prenatal care as well as the neonatal health and postpartum maternal outcomes. Controlling these numerous potential influences will be essential to inform current health (in)equity literature in the prenatal health care context (Braveman et al., 2000; Crear-Perry et al., 2021; Gadson et al., 2017). We assess adverse neonatal health outcomes like preterm birth, low birthweight (LBW), and neonatal intensive care unit (NICU) stays and postpartum maternal social support, depression symptomology, and confidence. These maternal outcomes are important to study due to their key role in new parents’ quality of life after delivery and the lasting impact on healthy child development (Albanese et al., 2019; Elsenbruch et al., 2007; Petterson & Albers, 2001).

The novelty and necessity of the current study finds a middle ground between the individual- and system-level. It explores how individual-level sociodemographic characteristics predict certain prenatal care experiences (i.e., responsive provider behavior, early entry into care, opting into group prenatal care programs) and, in turn, how those experiences predict several neonatal health and maternal postpartum outcomes. It also extends what is known about the long-term impact of prenatal care by going beyond infant health outcomes at birth and exploring associations of prenatal care that may persist into the postpartum period for women.

## Methods

### Participants and Procedures

The publicly available *Listening to Mothers III* dataset (Harris Interactive Inc., 2013) was used and is comprised of 2,400 online-survey participants (ages 18–45) who gave birth to singleton infants in hospitals between July 2011 and June 2012 in every U.S. state. The initial survey was conducted from October to December 2012 while the follow-up data were collected during January to April of 2013 with a 45% completion rate (*n* = 1072). Data were weighted by key demographic variables and the likelihood of the participants to be online. Thus, the *Listening to Mothers III* dataset uses sampling weights to be nationally representative. All descriptive, correlational, and structural equation analyses presented in this study used the appropriately weighted data to make inferences for the U.S. population. This study was deemed exempt by the university’s IRB as publicly available deidentified data are not human subjects research. For information and access to the *Listening to Mothers III* dataset, see https://dataverse.unc.edu/dataset.xhtml?persistentId=doi:10.15139/S3/11925.

### Measures

Variables assessed from the initial survey include all sociodemographic and pregnancy factors, prenatal care experience mediators, and neonatal health outcomes. The postpartum maternal wellbeing variables were assessed from the follow-up survey. Due to the timing of births and survey completions, follow-up question responses could have ranged from 6 to 21 months postpartum.

### Sociodemographic and Pregnancy Predictors

Age, race, income, insurance status, parous status, marital status, educational attainment, the perceived need of treatment for depression and help to quit smoking during recent pregnancy were identified as the key sociodemographic and pregnancy predictors. Each variable was dichotomized with the primary value coded as 1 (reference group coded as 0) and dummy coded when appropriate. Age was dummy coded to reflect ages 25–34 as the normative reference group, followed by young (18–24 years) and advanced mothers (35–45 years). Similarly, race was dummy coded to reflect White as the reference group, followed by Black, Hispanic/Latina, and Native and all others (i.e., Indigenous American, Alaskan Native, Native Hawaiian or other Pacific Islander, Asian, or some other race). Insurance was dummy coded to reflect private insurance as the reference group, followed by those who had some type of government coverage (i.e., Medicaid, CHIP, Tricare, Federal Employees Health Benefits, VA) and those who paid out of pocket. Parous status indicates those who were primiparous (i.e., first-time mothers) and marital status identifies those who were legally single at the time of the survey. Education level reflects those who received a high school education or less. Depression and smoking variables come from the survey items “during your recent pregnancy, did you feel you *needed* treatment for depression?” and “during your recent pregnancy, did you feel you *needed* help to quit smoking?”. Data were coded to reflect those who answered yes for needing treatment for depression and help to quit smoking.

### Prenatal Care Experience Mediators

Mediation variables include responsive provider behavior, the week of pregnancy that prenatal care was first initiated, and GPC attendance. Responsive provider behavior was created as an aggregate variable from five survey items (*a* = 0.72). The questions asked if the participant’s prenatal care provider “seemed rushed”, “used medical words you didn’t understand”, “spent enough time with you”, “answered all questions to your satisfaction”, and “encouraged you to talk about health questions and concerns”. Data from the five items were centered, standardized, and averaged to create a single continuous variable measuring provider behavior, with higher scores indicating more responsive behavior. The week of the first visit variable was continuous and was log transformed to account for the highly positive skewed nature of the variable. The variable of GPC was dichotomous.

### Neonatal Health and Postpartum Maternal Wellbeing Outcomes

Infant variables of gestational age (preterm; < 37 weeks), birthweight (LBW; < 5lbs 8oz), and NICU admittance (NICU stays; infant spent time in the NICU) were dichotomous. Postpartum maternal wellbeing variables were social support, depression, and confidence. Maternal social support was created as an aggregate variable that combined social support items from those who indicated support from a partner and those who indicated support from others. Items were scored on a five-point Likert scale and data were averaged and combined to produce a single continuous variable measuring overall social support (*a* = 0.90). The maternal depression variable was created by averaging and combining data from two items: “in the past two weeks, how often did you feel 1) little interest or pleasure in doing things and 2) feeling down, depressed or hopeless”. Answer options were on a four-point Likert scale and the resulting variable was continuous. Finally, maternal confidence was created from a single yes/no item: “Some women use the following words to describe their feelings in the weeks and months after birth. Thinking back to the first two months after you gave birth, did you feel confident?”. This item was part of a larger set of questions that were randomly assigned to only half of the sample.

### Analytic Plan

Descriptive and correlational analyses were run prior to the path analysis using structural equation modeling (SEM) with Full Information Maximum Likelihood (FIML) in Stata SE17. A robust estimator was used to avoid the assumption of normally distributed errors. Two separate path models were run that differed only by outcomes: a neonatal health model and a postpartum maternal wellbeing model. The models included pathways from predictors to both mediators and outcomes, and analyses factored in weighting of the data. Results reported in the text and in figures are those that reached a significance level of *p* <.01.

Missing data was accounted for using FIML. This provides unbiased estimates under the assumption that data are missing at random and includes the full sample. Given the nature of the two time-point study design, attrition did occur. From the initial sample of 2400 participants, 1072 (45%) completed the follow-up survey. Low-income women and Black women were the most likely to attrit from the study, with increases in the odds of 107% and 99%, respectively, compared to non-low-income and White women. Given the levels of selective attrition in the sample, it is important to employ FIML to produce less biased parameter estimates related to potential selection effects of who remains in the study (Acock, 2012).

## Results

See Tables 1 and 2, respectively, for descriptive and correlational analyses. All participants identified as female with ages ranging between 18 and 45 years (*M* = 29.16, *SD* = 6.05). Nearly 38% of the sample had incomes below 200% of the poverty line. Primiparous (i.e., first-time) mothers made up 41% of the sample while unmarried mothers made up 39%. Twenty-two percent of the sample indicated they participated in GPC. Week of first prenatal care visit averaged 8.41 weeks (*SD* = 4.78 weeks) and ranged from 2 to 40 weeks. With data being first centered and standardized, and then weighted, responsive provider behavior had an average score of -0.04 with a standard deviation of 1.03. Of infants in the sample, 8% were born preterm, 8% were born LBW, and 18% were admitted into the NICU. An increase in NICU stays was significantly correlated with mother attendance at GPC (*r* =.30, *p* <.001) but a decrease in NICU stays was significantly correlated with more responsive provider behavior (*r* = −.14, *p* <.001). For postpartum maternal wellbeing, social support averaged scores of 3.61 (*SD* = 0.95) on a scale of 1–5 while depression averaged scores of 1.55 (*SD* = 0.81) on a scale of 1–4. Seventy-two percent of mothers indicated they felt confident in the two months after delivery. Responsive provider behavior was significantly correlated with each postpartum maternal wellbeing outcome: social support (*r* =.25, *p* <.001), depression (*r* = −.23, *p* <.001), and confidence (*r* =.11, *p* =.009).

**Table 1:** Descriptive statistics of weighted model variables

Variable	*N*	M or %	SD	Range
**Sociodemographic and Pregnancy Factors**				
Age				
Normative (ages 25–34)	1274.40	53.10%		
Young (ages 18–24)	762.96	31.79%		
Advanced (ages 35–45)	362.64	15.11%		
Race/Ethnicity				
White	1308.00	54.50%		
Black	368.16	15.34%		
Hispanic/Latina	555.36	23.14%		
Native & All Others	168.48	7.02%		
Income				
< 200% Federal Poverty Line	905.86	37.74%		
> 200% Federal Poverty Line	1358.07	56.59%		
Missing	136.06	5.67%		
Insurance				
None/Out of Pocket	119.42	4.98%		
Governmental	1117.18	46.55%		
Private	1091.38	45.47%		
Missing	72.02	3.00%		
Parous Status				
Primiparous	977.52	40.73%		
Perceived Need of Treatment for:				
Depression	369.36	15.39%		
Help to Quit Smoking	267.84	11.16%		
Marital Status				
Unmarried	931.32	38.80%		
Married	1450.51	60.44%		
Missing	18.17	0.76%		
Education Level				
High School Degree or Less	1014.00	42.25%		
**Prenatal Care Experience Mediators**				
Group Prenatal Care	539.76	22.49%		
Responsive Provider Behavior	2400.00	-0.04	1.03	-3.53-1.13
Week of First Care Visit	2400.00	8.41	4.78	2–40
**Neonatal Health**				
Low Birthweight	187.68	7.82%		
Gestationally Preterm	196.80	8.20%		
NICU Status				
Admitted (NICU Stay)	426.51	17.77%		
Not Admitted	1925.38	80.22%		
Missing	48.11	2.00%		
**Postpartum Maternal Wellbeing**				
Maternal Social Support	983.11	3.61	0.95	1–5
Missing	1416.89			
Maternal Depression	981.05	1.55	0.81	1–4
Missing	1418.95			
Maternal Confidence	484.19	71.75%		
Missing	1915.81			
Total	2400.00			

Note: The statistics shown here used a sampling weight to make them nationally representative

**Table 2:** Correlational data for weighted model variables

	1.	2.	3.	4.	5.	6.	7.	8.	9.	10.
1. LBW	-									
2. Preterm	0.29***	-								
3. NICU Stay	0.33***	0.31***	-							
4. Maternal Social Support	-0.02	-0.10***	-0.07*	-						
5. Maternal Depression	0.10**	0.03	0.07*	-0.27***	-					
6. Maternal Confidence	-0.02	-0.03	0.07^†^	0.21***	-0.15***	-				
7. Responsive Provider Behavior	-0.05*	-0.05*	-0.14***	0.24***	-0.23***	0.11**	-			
8. Group Prenatal Care	0.13***	-0.03	0.30***	0.14***	0.13***	0.14**	-0.20***	-		
9. Week of First Visit	-0.10***	0.02	-0.06**	-0.13***	-0.04	-0.05	-0.04^†^	-0.12***	-	
10. Duration of Visits	0.03	0.01	-0.01	0.07*	0.07*	-0.05	-0.04*	0.10***	0.01	-
11. Age– Normative	-0.10***	0.03	0.07**	0.03	-0.13***	0.11*	0.04^†^	-0.14***	-0.02	-0.08***
12. Age– Young	0.06**	-0.06**	0.10***	0.01	0.12***	0.11**	-0.14***	0.20***	0.02	0.16***
13. Age– Advanced	0.06**	0.03^†^	-0.03	-0.04	0.04	0.02	0.13***	-0.07**	0.00	-0.10***
14. Race/Ethnicity– White	-0.05*	0.01	-0.12***	-0.04	-0.05	-0.08^†^	0.04*	-0.16***	0.05*	-0.13***
15. Race/Ethnicity– Black	0.09***	0.02	0.04*	0.15***	-0.08*	0.11*	0.05*	0.08***	-0.07***	0.10***
16. Race/Ethnicity– Hispanic/Latina	-0.05*	-0.01	0.05*	-0.06*	0.07*	-0.03	-0.06**	0.06**	0.03	0.04*
17. Race/Ethnicity– Native & Others	0.05**	-0.02	0.09***	-0.01	0.09**	0.06	-0.06**	0.09***	-0.04*	0.03
18. Income– 200% Below Poverty	-0.02	0.04*	-0.04*	-0.18***	0.05^†^	-0.05	0.00	-0.11***	0.06**	0.09***
19. Insurance– None/Out of Pocket	0.07**	-0.03	0.05**	-0.03	0.12***	-0.01	-0.11***	0.21***	-0.01	0.04^†^
20. Insurance– Governmental	0.03	0.04^†^	0.07***	-0.10**	0.06*	0.00	-0.03	0.01	0.08***	0.13***
21. Insurance– Private	-0.06**	-0.02	-0.10***	0.12***	-0.11***	0.00	0.08***	-0.10***	-0.08***	-0.15***
22. Parous Status– Primiparous	0.05*	-0.06**	0.11***	0.11***	0.04	-0.05	-0.07***	0.19***	-0.09***	0.04*
23. In Need of Treatment: Depression	0.07**	0.04*	0.26***	-0.07*	0.21***	0.00	-0.24***	0.21***	0.00	0.07***
24. In Need of Treatment: Smoking	0.12***	0.02	0.28***	0.01	0.13***	0.04	-0.16***	0.27***	-0.01	0.03
25. Marital Status– Unmarried	0.12***	-0.03	0.04*	-0.02	0.10***	0.01	-0.09***	0.06**	0.02	0.16***
26. Education– High School or Less	0.02	0.03^†^	0.02	-0.13***	0.07*	0.03	-0.09***	0.01	0.07***	0.04*

Note: ^†^*p* <.10, *p <.05, **p <.01, ***p <.001

### Neonatal Health Model

**Fig. 1:**
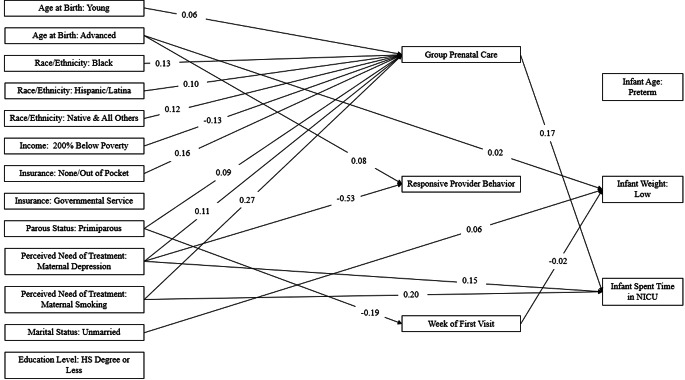
Associations between Sociodemographic and Pregnancy Factors, Prenatal Care Mediators, and Neonatal Health Outcomes *Note*: Direct effect values limited to those significant at *p* <.01 for the sake of figure legibility. For all other associations, see Tables S2 and S3

For the neonatal health model, see Fig. 1 for direct associations significant at *p* <.01. Notably, direct effects of perceiving a need of treatment for depression or smoking during pregnancy indicated that the proportion of infants in NICU were 0.15 and 0.20 larger for these groups, respectively, compared to their counterparts. Unlike NICU stays, maternal sociodemographic and pregnancy characteristics had fewer associations with LBW. Only women of advanced age (*b* = 0.02, *p* =.004) and unmarried women (*b* = 0.06, *p* =.002) had direct effects relating to a greater proportion of infants born LBW.

Contrary to previous literature, involvement in GPC was associated with a proportion increase of 0.17 for infants admitted to the NICU. However, GPC was also most significantly related to maternal sociodemographic and pregnancy factors, which indicates that, overall, these pregnant women have higher rates of involvement with GPC than their counterparts. Additionally, regardless of type of prenatal care, one standard deviation of later entry into prenatal care related to a proportion decrease of 0.02 for infants born LBW (*p* =.009). Finally, women of advanced age were more likely to report responsive provider behavior (*b* = 0.08, *p* <.001), while women who needed treatment for depression during pregnancy were less likely to report the same (*b* = -0.53, *p* <.001).

### Postpartum Maternal Wellbeing Model

**Fig. 2:**
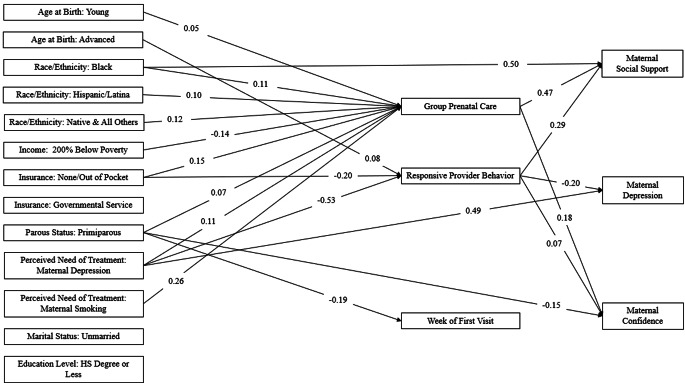
Associations between Sociodemographic and Pregnancy Factors, Prenatal Care Mediators, and Postpartum Maternal Wellbeing Outcomes Note: Direct effect values limited to those significant at *p* <.01 for the sake of figure legibility. For all other associations, see Tables S4 and S5

See Fig. 2 for direct associations significant at *p* <.01. Results showed that Black women were significantly more likely to report being socially supported both directly (*b* = 0.50, *p* <.001) and through an indirect prenatal care mediation (*b* = 0.11, *p* =.003). Additionally, advanced maternal age resulted in an overall indirect effect on depression in the postpartum (-0.02, *p* =.005), meaning that advanced age is related to lower reports of depressive symptomology mediated by prenatal care experiences. Primiparous women were more likely to indicate feeling less confident than their multiparous counterparts (*b* = -0.15, *p* =.005). Black women, through an overall mediation effect, reported feeling more confident than their White counterparts (*b* = 0.03, *p* =.007).

Notably, women who perceived their provider behavior as responsive had higher rates of social support (*b* = 0.29, *p* <.001), reported less depression (*b* = -0.20, *p* <.001), and felt more confident (*b* = 0.07, *p* =.005; see Table S5). Women involved with GPC also indicated more social support (*b* = 0.47, *p* <.001) and had an 0.18 larger proportion of women feeling confident (*p* <.001). Women of advanced age reported more responsive behavior (*b* = 0.08, *p* <.001) while those who paid out of pocket for care (*b* = -0.20, *p* =.007) or had a perceived need of treatment for depression (*b* = -0.53, *p* <.001) reported less responsive provider behavior. Finally, results indicated that primiparous women initiated prenatal care earlier than their counterparts (*b* = -0.19, *p* =.004). For all other neonatal health and postpartum maternal wellbeing SEM associations (i.e., *p* <.05, trending *p* <.10, or non-significant; direct, indirect, and total effects), see Tables S1-S5 at https://osf.io/2py9z/?view_only=e3a295acdff34426be51a6894d564035.

## Discussion

Results indicated that prenatal care experiences consist of both positive and negative associations concerning the health and wellbeing of infants and their mothers. Overall, individual maternal characteristics related more to neonatal health outcomes but interpersonal experiences during prenatal care related more to postpartum maternal wellbeing outcomes. Only GPC attendance was significantly associated with both, where participating in GPC was associated with increases in the proportion of infants being admitted to the NICU but was also significantly associated with increases in postpartum maternal social support. Another salient finding was that responsive provider behavior was significantly associated with all the postpartum maternal wellbeing outcomes measured here (i.e., social support, depression, and confidence).

### Key Contributions and Implications

Surprisingly, the neonatal health model demonstrated little evidence of prenatal care experiences being protective. Instead, this study offers important information for future research to explore important areas of improvement in prenatal care regarding women and their infants. As such, several predictors were identified as most strongly related to key neonatal health outcomes. In the neonatal health model, NICU stays had the most numerous and significant direct and indirect effects compared to other outcomes. Particularly, while controlling for all other factors, direct effects indicated that perceived needs of treatment for smoking and depression, and involvement in GPC resulted in an increased risk for NICU stays. The first finding is supported in previous literature relating to adverse infant birth outcomes being predicted by prenatal smoking behaviors (Ratnasiri et al., 2020) while the latter two are not. Where literature was mixed about the relation between maternal depression and adverse infant birth outcomes (Ecklund-Flores et al., 2017; Grigoriadis et al., 2013), this study contributed findings to support the connection. Additionally, the positive association between GPC and NICU stays was in opposition to findings that indicated GPC either lessens the risk of adverse infant health (Crockett et al., 2019; Ickovics et al., 2007; Thielen, 2012) or does not statistically differ in NICU admissions from individual prenatal care (Carter et al., 2016). Research is still needed to determine which factors contribute to the health disparities related to these populations of women and why GPC continues to see mixed findings of infant health.

Unlike the neonatal health model, GPC was promotive of both maternal social support and confidence in the postpartum maternal wellbeing model as predicted (Chae et al., 2017; Cunningham et al., 2017). Although differences in more objective (i.e., neonatal health) versus more subjective (i.e., postpartum maternal wellbeing) experiences may be at play, these contradictory results between models beg for a more nuanced approach to understanding GPC and its potential effects. Additionally, responsive provider behavior was more predictive of postpartum maternal wellbeing compared to neonatal health– higher reports of responsive provider behavior predicted higher social support, lower depression, and higher confidence. This aligns with a recent evidence-based review of medical interventions suggesting that if patient-provider interactions are improved, the patient benefits from improved health and care satisfaction (Drossman et al., 2021). Here, prenatal care experiences showed evidence of being protective with responsive provider behavior having significant associations across all postpartum wellbeing outcomes. Still, there was evidence of less responsive provider behavior toward those who were uninsured and who perceived a need for treatment for depression during pregnancy, aligning with prior findings of discrimination in prenatal care (Ward et al., 2013).

Depression and smoking during pregnancy resulted in some of the strongest and most concerning findings relating maternal characteristics to neonatal health. Fortunately, these factors are malleable and potentially easier to intervene upon during the prenatal care period as opposed to intervening on broader systemic issues (i.e., social determinants of health) that contribute to health inequity. This study provides an opportunity for prenatal care providers to reassess the ways in which they address maternal mental health and health behaviors during pregnancy.

### Strengths, Limitations, and Future Directions

One strength of this study included the identification of specific, interpersonal prenatal care experiences that were most related to neonatal health and postpartum maternal wellbeing. This study simultaneously included many sociodemographic and pregnancy predictors that allowed for more precise estimates while controlling potential confounds. Additionally, this large, nationally representative study was able to capture data from two key timepoints and allowed for the examination of associations enduring beyond birth into the postpartum period.

Limitations include those inherent to secondary data analyses. Notably, all variables were retrospective maternal reports. Although still valuable, subjective perceptions must be interpreted with caution. Next, because the predictor variables were dichotomous, data lacked the nuance of the identities of an individual’s experiences. Further, the prenatal care variable of GPC was not robust in its measurement of prenatal care type. It was simply a measure of if a sample participant had ever attended at least one group session, which left much unaddressed (e.g., dosage/mixed attendance with individual and group care; GPC program type provided by clinic, quality of GPC program delivery). A final limitation is the age of the data. However, it could be argued that the conditions around maternal and infant health are as similar in severity, if not worse, than they were a decade ago (March of Dimes, 2024). For example, 8% of infants were born preterm and LBW during this study conducted in 2011–2012 and yet the rates had increased to 10.4% and 8.6% for the same respective outcomes a decade later (National Center for Health Statistics, 2024). Admittance to the NICU and both infant and maternal mortality have also tended to increase each year since the *Listening to Mothers III* study was made available (March of Dimes, 2024; Martin & Osterman, 2025). Therefore, the data and its insights remain valuable.

With significant associations across postpartum maternal wellbeing, responsive provider behavior and GPC were salient factors in this study and present opportunities for future research. Pregnant individuals have different medical (e.g., health conditions/complications) and support needs (e.g., physician/care preferences) during pregnancy (Peahl et al., 2020) that could be explored in relation to patient perceptions of quality care and resulting health outcomes. Moreover, the influence of prenatal care experiences on postpartum outcomes is compelling. How does a relatively isolated experience with prenatal care across nine months or less impact the wellbeing and support of parents beyond infant delivery? Future work could assess the impact of other social determinants of health and broader upstream factors related to prenatal care, infant health, and postpartum maternal wellbeing. For example, housing insecurity is related to both inadequate prenatal care utilization and adverse infant outcomes (DiTosto et al., 2021). Considering the potential for overlapping racial or socioeconomic disparities (Reece, 2021), addressing the issue of safe housing or other social determinants of health may be an even more crucial first step in promoting health equity.

Prenatal care programs or maternal and child health related policies can also help promote equitable perinatal outcomes. The GPC program of CenteringPregnancy (Rising, 1998) has demonstrated positive associations with neonatal health (Crockett et al., 2019; Ickovics et al., 2008) and postpartum wellbeing (Chae et al., 2017; Cunningham et al., 2017). This is especially encouraging given that this type of group care also tends to appeal to pregnant individuals who are younger in age, single, first-time parents, and/or belonging to a marginalized racial identity (Cunningham et al., 2017; Picklesimer et al., 2012). Additionally, widespread access to health insurance and quality prenatal care (e.g., Medicaid expansion, doula reimbursement, culturally competent care, reducing maternity care deserts) would contribute to improved neonatal and postpartum outcomes (Black Maternal Health Federal Policy Collective, 2025; March of Dimes, 2018). Packaged legislation like the successfully passed S.B. 65: California Momnibus Act (Office of Governor Gavin Newson, 2021) and the previously introduced S.1606 Black Maternal Health Momnibus Act (118th Congress, 2023) are worthwhile state- and federal-level avenues for bettering the health of infants, birthing parents, and families.

## Conclusions

While prenatal care is often thought about in terms of promoting infant health, the prenatal care experiences in this study were not associated with improved neonatal outcomes. Instead, it was the postpartum maternal wellbeing outcomes that were positively related to the prenatal care experiences– specifically responsive provider behavior and group prenatal care. For providers and prenatal care practices, this is a crucial reframing in how the experiences during prenatal care extend beyond birth and delivery of infants and how the interpersonal relationships and support resources remain important for parents even after care has ended.

Findings from this study suggest two actions prenatal care practices could take that would be most impactful for pregnant populations. The first would be to provide increased support to those who identify as needing treatment for depression or smoking during pregnancy to best improve neonatal health through fewer admissions into the NICU. The second would be to continue to bolster the responsiveness in which providers interact with patients to improve overall postpartum maternal wellbeing. Based on the mixed findings of GPC on neonatal health and postpartum wellbeing outcomes in this study, it is further suggested that additional and more nuanced research on these group programs is done to ensure equitable care and outcomes for both infants and parents.

## Electronic Supplementary Material

Below is the link to the electronic supplementary material.

Supplementary Material 1

## Data Availability

For information and access to the *Listening to Mothers III* dataset, see https://dataverse.unc.edu/dataset.xhtml?persistentId=doi:10.15139/S3/11925.

## Code Availability

Analytic code and supplementary tables and figures are available at https://osf.io/2py9z/?view_only=e3a295acdff34426be51a6894d564035.
