# Supplementary material for: Maternal Characteristics and U.S. Prenatal Care: Associations with Neonatal Health and Postpartum Maternal Wellbeing
Source: Matern Child Health J. 2025 Jul 7;29(9):1232–43. doi: 10.1007/s10995-025-04128-0 (PMC12460427; doi:10.1007/s10995-025-04128-0)
Supplement: Supplementary file 1 — Supplementary Material 1 [file 10995_2025_4128_MOESM1_ESM.docx]

Maternal Characteristics and U.S. Prenatal Care:

Associations with Neonatal Health and Postpartum Maternal Wellbeing

Maternal and Child Health Journal

Inga Nordgren, MS^1^, Robert J. Duncan, PhD^1^, Kameron J. Moding, PhD^1^, German E. Posada, PhD^1^

Purdue University, Department of Human Development and Family Science^1^

Email of corresponding author: inordgre@purdue.edu

**Table S1**

***Structural Equation Values for Neonatal Health Model***

|  | Direct Effects | | Indirect Effects | | Total Effects | |
| --- | --- | --- | --- | --- | --- | --- |
| **Infant Outcomes** | Estimate | *SE* | Estimate | *SE* | Estimate | *SE* |
| *Preterm* |  |  |  |  |  |  |
| Age – Young | -0.02 | 0.01 | 0.00 | 0.00 | -0.02 | 0.01 |
| Age – Advanced | 0.01 | 0.01 | 0.00 | 0.00 | 0.01 | 0.01 |
| Race/Ethnicity – Black | 0.02 | 0.03 | 0.01 | 0.00 | 0.02 | 0.03 |
| Race/Ethnicity – Hispanic/Latina | -0.01 | 0.02 | 0.00 | 0.00 | -0.01 | 0.02 |
| Race/Ethnicity – Native & All Others | -0.02 | 0.03 | 0.00 | 0.00 | -0.02 | 0.03 |
| Income – 200% Below Poverty | 0.02 | 0.02 | 0.00 | 0.00 | 0.02 | 0.02 |
| Insurance – None/Out of Pocket | -0.01 | 0.01 | 0.00 | 0.00 | -0.01 | 0.01 |
| Insurance – Governmental | 0.01 | 0.02 | 0.00 | 0.00 | 0.01 | 0.02 |
| Parous Status – Primiparous | -0.01 | 0.02 | 0.00 | 0.00 | -0.01 | 0.02 |
| In Need of Treatment for Depression | 0.03 | 0.03 | 0.01 | 0.01 | 0.04 | 0.03 |
| In Need of Treatment for Smoking | 0.01 | 0.03 | 0.00 | 0.01 | 0.01 | 0.03 |
| Marital Status – Unmarried | -0.02 | 0.02 | 0.00 | 0.00 | -0.02 | 0.02 |
| Education – High School or Less | 0.02 | 0.02 | 0.00 | 0.00 | 0.02 | 0.02 |
| Group Prenatal Care | -0.02 | 0.02 |  |  |  |  |
| Responsive Provider Behavior | -0.02 | 0.01 |  |  |  |  |
| Week of First Visit | 0.00 | 0.01 |  |  |  |  |
| *Low Birthweight* |  |  |  |  |  |  |
| Age – Young | 0.00 | 0.01 | 0.00 | 0.00 | 0.00 | 0.01 |
| Age – Advanced | 0.02** | 0.01 | 0.00 | 0.00 | 0.02** | 0.01 |
| Race/Ethnicity – Black | 0.03 | 0.03 | 0.01^†^ | 0.01 | 0.04 | 0.03 |
| Race/Ethnicity – Hispanic/Latina | -0.02 | 0.02 | 0.01^†^ | 0.00 | -0.02 | 0.02 |
| Race/Ethnicity – Native & All Others | 0.04 | 0.04 | 0.01^†^ | 0.01 | 0.05 | 0.04 |
| Income – 200% Below Poverty | -0.03 | 0.02 | -0.01^†^ | 0.00 | -0.03 | 0.02 |
| Insurance – None/Out of Pocket | 0.03 | 0.03 | 0.01 | 0.00 | 0.04 | 0.03 |
| Insurance – Governmental | 0.03 | 0.02 | 0.00 | 0.00 | 0.02 | 0.02 |
| Parous Status – Primiparous | 0.01 | 0.02 | 0.01* | 0.00 | 0.01 | 0.02 |
| In Need of Treatment for Depression | 0.01 | 0.03 | 0.01 | 0.01 | 0.01 | 0.03 |
| In Need of Treatment for Smoking | 0.06 | 0.04 | 0.01* | 0.01 | 0.08^†^ | 0.04 |
| Marital Status – Unmarried | 0.06** | 0.02 | 0.00 | 0.00 | 0.06** | 0.02 |
| Education – High School or Less | 0.00 | 0.02 | 0.00 | 0.00 | 0.00 | 0.02 |
| Group Prenatal Care | 0.05* | 0.02 |  |  |  |  |
| Responsive Provider Behavior | 0.00 | 0.01 |  |  |  |  |
| Week of First Visit | -0.02** | 0.01 |  |  |  |  |
| *NICU Stay* |  |  |  |  |  |  |
| Age – Young | 0.01 | 0.01 | 0.01** | 0.00 | 0.02 | 0.03 |
| Age – Advanced | 0.01 | 0.01 | 0.00 | 0.00 | 0.00 | 0.01 |
| Race/Ethnicity – Black | 0.05 | 0.03 | 0.02* | 0.01 | 0.07* | 0.03 |
| Race/Ethnicity – Hispanic/Latina | 0.05^†^ | 0.03 | 0.02*** | 0.01 | 0.07* | 0.03 |
| Race/Ethnicity – Native & All Others | 0.10* | 0.05 | 0.03** | 0.01 | 0.13* | 0.05 |
| Income – 200% Below Poverty | -0.05^†^ | 0.03 | -0.03*** | 0.01 | -0.07** | 0.03 |
| Insurance – None/Out of Pocket | 0.00 | 0.03 | 0.03*** | 0.01 | 0.03 | 0.03 |
| Insurance – Governmental | 0.05^†^ | 0.03 | 0.01 | 0.01 | 0.06^†^ | 0.03 |
| Parous Status – Primiparous | 0.04^†^ | 0.02 | 0.02** | 0.01 | 0.06* | 0.02 |
| In Need of Treatment for Depression | 0.15*** | 0.04 | 0.03** | 0.01 | 0.18*** | 0.04 |
| In Need of Treatment for Smoking | 0.20*** | 0.05 | 0.05*** | 0.01 | 0.25*** | 0.05 |
| Marital Status – Unmarried | -0.03 | 0.02 | -0.01 | 0.01 | -0.04 | 0.03 |
| Education – High School or Less | 0.01 | 0.03 | 0.00 | 0.01 | 0.01 | 0.03 |
| Group Prenatal Care | 0.17*** | 0.03 |  |  |  |  |
| Responsive Provider Behavior | -0.02^†^ | 0.01 |  |  |  |  |
| Week of First Visit | -0.01 | 0.01 |  |  |  |  |

Note: ^†^*p* < .10, **p* < .05, ***p* < .01, ****p* < .001.

**Table S2**

***Direct Associations between Maternal Sociodemographic and Pregnancy Factors with Prenatal Care Experience Mediators in Neonatal Health SEM***

|  | Group Prenatal Care | | Responsive Provider Behavior | | Week of First Visit | |
| --- | --- | --- | --- | --- | --- | --- |
|  | Estimate | *SE* | Estimate | *SE* | Estimate | *SE* |
| Age – Young | 0.06*** | 0.02 | -0.08^†^ | 0.04 | 0.04 | 0.04 |
| Age – Advanced | 0.00 | 0.01 | 0.08*** | 0.02 | 0.00 | 0.03 |
| Race/Ethnicity – Black | 0.13*** | 0.04 | 0.15^†^ | 0.09 | -0.29* | 0.12 |
| Race/Ethnicity – Hispanic/Latina | 0.10*** | 0.03 | -0.07 | 0.08 | -0.07 | 0.08 |
| Race/Ethnicity – Native & All Others | 0.12** | 0.04 | -0.11 | 0.12 | -0.20 | 0.13 |
| Income – 200% Below Poverty | -0.13*** | 0.03 | 0.11 | 0.08 | 0.03 | 0.09 |
| Insurance – None/Out of Pocket | 0.16*** | 0.03 | -0.19* | 0.07 | 0.04 | 0.08 |
| Insurance – Governmental | 0.04 | 0.03 | 0.00 | 0.08 | 0.16^†^ | 0.08 |
| Parous Status – Primiparous | 0.09*** | 0.03 | -0.01 | 0.06 | -0.19** | 0.07 |
| In Need of Treatment for Depression | 0.11** | 0.04 | -0.53*** | 0.10 | -0.02 | 0.13 |
| In Need of Treatment for Smoking | 0.27*** | 0.05 | -0.21^†^ | 0.12 | -0.05 | 0.15 |
| Marital Status – Unmarried | -0.04 | 0.03 | -0.07 | 0.07 | 0.03 | 0.09 |
| Education Level – HS Degree or Less | 0.00 | 0.03 | -0.11 | 0.07 | 0.07 | 0.08 |

Note: ^†^*p* < .10, **p* < .05, ***p* < .01, ****p* < .001.

**Table S3**

***Structural Equation Values for Postpartum Maternal Wellbeing Model***

|  | Direct Effects | | Indirect Effects | | Total Effects | |
| --- | --- | --- | --- | --- | --- | --- |
| **Postpartum Wellbeing Outcomes** | Estimate | *SE* | Estimate | *SE* | Estimate | *SE* |
| *Maternal Social Support* |  |  |  |  |  |  |
| Age – Young | 0.02 | 0.06 | 0.00 | 0.02 | 0.02 | 0.07 |
| Age – Advanced | -0.06 | 0.04 | 0.02** | 0.01 | -0.04 | 0.04 |
| Race/Ethnicity – Black | 0.50*** | 0.13 | 0.11** | 0.04 | 0.61*** | 0.13 |
| Race/Ethnicity – Hispanic/Latina | 0.15 | 0.15 | 0.03 | 0.03 | 0.18 | 0.15 |
| Race/Ethnicity – Native & All Others | -0.03 | 0.21 | 0.04 | 0.04 | 0.01 | 0.21 |
| Income – 200% Below Poverty | -0.32* | 0.13 | -0.04 | 0.03 | -0.36** | 0.14 |
| Insurance – None/Out of Pocket | -0.18 | 0.16 | 0.01 | 0.04 | -0.17 | 0.15 |
| Insurance – Governmental | -0.27* | 0.13 | -0.01 | 0.03 | -0.27* | 0.13 |
| Parous Status – Primiparous | 0.11 | 0.09 | 0.04 | 0.02 | 0.15 | 0.10 |
| In Need of Treatment for Depression | -0.26^†^ | 0.16 | -0.11* | 0.05 | -0.37* | 0.16 |
| In Need of Treatment for Smoking | 0.16 | 0.21 | 0.06 | 0.05 | 0.23 | 0.20 |
| Marital Status – Unmarried | -0.02 | 0.12 | 0.03 | 0.05 | 0.02 | 0.14 |
| Education – High School or Less | -0.13 | 0.12 | -0.04^†^ | 0.02 | -0.18 | 0.12 |
| Group Prenatal Care | 0.47*** | 0.13 |  |  |  |  |
| Responsive Provider Behavior | 0.29*** | 0.05 |  |  |  |  |
| Week of First Visit | -0.07 | 0.05 |  |  |  |  |
| *Maternal Depression* |  |  |  |  |  |  |
| Age – Young | 0.07 | 0.07 | 0.02 | 0.01 | 0.08 | 0.07 |
| Age – Advanced | 0.10* | 0.04 | -0.02** | 0.01 | 0.08* | 0.04 |
| Race/Ethnicity – Black | -0.35* | 0.16 | -0.01 | 0.02 | -0.36* | 0.15 |
| Race/Ethnicity – Hispanic/Latina | -0.05 | 0.14 | 0.02 | 0.02 | -0.03 | 0.14 |
| Race/Ethnicity – Native & All Others | 0.21 | 0.23 | 0.03 | 0.03 | 0.24 | 0.24 |
| Income – 200% Below Poverty | 0.07 | 0.12 | -0.02 | 0.03 | 0.05 | 0.13 |
| Insurance – None/Out of Pocket | 0.29 | 0.22 | 0.04 | 0.03 | 0.33 | 0.20 |
| Insurance – Governmental | 0.14 | 0.13 | 0.00 | 0.02 | 0.14 | 0.13 |
| Parous Status – Primiparous | -0.02 | 0.09 | 0.01 | 0.02 | 0.00 | 0.09 |
| In Need of Treatment for Depression | 0.49** | 0.19 | 0.11* | 0.04 | 0.60*** | 0.18 |
| In Need of Treatment for Smoking | 0.10 | 0.22 | 0.05 | 0.05 | 0.15 | 0.22 |
| Marital Status – Unmarried | 0.26^†^ | 0.14 | -0.01 | 0.03 | 0.24^†^ | 0.14 |
| Education – High School or Less | -0.03 | 0.11 | 0.02 | 0.02 | -0.01 | 0.12 |
| Group Prenatal Care | 0.02 | 0.15 |  |  |  |  |
| Responsive Provider Behavior | -0.20*** | 0.06 |  |  |  |  |
| Week of First Visit | -0.03 | 0.05 |  |  |  |  |
| *Maternal Confidence* |  |  |  |  |  |  |
| Age – Young | 0.07^†^ | 0.04 | 0.00 | 0.01 | 0.07* | 0.04 |
| Age – Advanced | 0.00 | 0.02 | 0.01* | 0.00 | 0.00 | 0.02 |
| Race/Ethnicity – Black | 0.13* | 0.07 | 0.03** | 0.01 | 0.17* | 0.07 |
| Race/Ethnicity – Hispanic/Latina | -0.01 | 0.07 | 0.01 | 0.01 | 0.00 | 0.07 |
| Race/Ethnicity – Native & All Others | 0.15* | 0.07 | 0.02 | 0.01 | 0.16* | 0.07 |
| Income – 200% Below Poverty | -0.06 | 0.06 | -0.02^†^ | 0.01 | -0.08 | 0.06 |
| Insurance – None/Out of Pocket | -0.04 | 0.06 | 0.01 | 0.01 | -0.02 | 0.06 |
| Insurance – Governmental | -0.05 | 0.06 | 0.00 | 0.01 | -0.05 | 0.06 |
| Parous Status – Primiparous | -0.15** | 0.05 | 0.01 | 0.01 | -0.13* | 0.05 |
| In Need of Treatment for Depression | -0.08 | 0.07 | -0.02 | 0.02 | -0.10 | 0.07 |
| In Need of Treatment for Smoking | -0.02 | 0.09 | 0.03^†^ | 0.02 | 0.02 | 0.09 |
| Marital Status – Unmarried | 0.03 | 0.07 | 0.01 | 0.01 | 0.04 | 0.07 |
| Education – High School or Less | 0.03 | 0.07 | -0.01 | 0.01 | 0.02 | 0.07 |
| Group Prenatal Care | 0.18*** | 0.06 |  |  |  |  |
| Responsive Provider Behavior | 0.07** | 0.02 |  |  |  |  |
| Week of First Visit | -0.01 | 0.02 |  |  |  |  |

Note: ^†^*p* <0.10, **p* < .05, ***p* < .01, ****p* < .001.

**Table S4**

***Associations between Maternal Sociodemographic and Pregnancy Factors with Prenatal Care Experience Mediators in Postpartum Maternal Wellbeing SEM***

|  | Group Prenatal Care | | Responsive Provider Behavior | | Week of First Visit | |
| --- | --- | --- | --- | --- | --- | --- |
|  | Estimate | *SE* | Estimate | *SE* | Estimate | *SE* |
| Age – Young | 0.05** | 0.02 | -0.08* | 0.04 | 0.04 | 0.05 |
| Age – Advanced | 0.00 | 0.01 | 0.08*** | 0.02 | 0.00 | 0.03 |
| Race/Ethnicity – Black | 0.11** | 0.04 | 0.13 | 0.09 | -0.29* | 0.12 |
| Race/Ethnicity – Hispanic/Latina | 0.10*** | 0.03 | -0.07 | 0.08 | -0.07 | 0.09 |
| Race/Ethnicity – Native & All Others | 0.12** | 0.04 | -0.11 | 0.11 | -0.20 | 0.13 |
| Income – 200% Below Poverty | -0.14*** | 0.03 | 0.09 | 0.08 | 0.02 | 0.09 |
| Insurance – None/Out of Pocket | 0.15*** | 0.03 | -0.20** | 0.07 | 0.04 | 0.08 |
| Insurance – Governmental | 0.03 | 0.03 | -0.02 | 0.08 | 0.17* | 0.08 |
| Parous Status – Primiparous | 0.07** | 0.03 | -0.02 | 0.06 | -0.19** | 0.07 |
| In Need of Treatment for Depression | 0.11** | 0.04 | -0.53*** | 0.10 | -0.01 | 0.13 |
| In Need of Treatment for Smoking | 0.26*** | 0.05 | -0.22^†^ | 0.12 | -0.06 | 0.15 |
| Marital Status – Unmarried | 0.05 | 0.06 | 0.05 | 0.13 | 0.05 | 0.16 |
| Education Level – HS Degree or Less | -0.01 | 0.03 | -0.12^†^ | 0.07 | 0.07 | 0.08 |

Note: ^†^*p* < .10, **p* < .05, ***p* < .01, ****p* < .001
